# Increasing the Light Extraction Efficiency of Organic Light-Emitting Devices by Electrochemically Corroded Patterned Substrates

**DOI:** 10.3390/mi15010067

**Published:** 2023-12-29

**Authors:** Yang Wang, Zhonghao Li, Yu Bai, Yingzhi Wang

**Affiliations:** School of Electronic Information Engineering, Changchun University of Science and Technology, Changchun 130022, China

**Keywords:** OLED, silicon substrate, electrochemically corroded

## Abstract

A substrate with microstructure can increase the light extraction efficiency of OLEDs. However, the present preparation methods for micro- and nanostructures are not suited for broad-area manufacturing. In this research, we suggested an electrochemical etching approach to patterning Si substrates and effectively generated a vast area of micro-/nanostructures on the surface of Si. We created OLEDs using this patterned substrate. It was discovered through this study that when the current density is 100 mA/cm^2^, the brightness increases by 1.67 times and the efficiency increases by 1.43 times, over a planar equivalent. In the future, this electrochemical etching process for patterned silicon substrates might give rise to a new approach to the large-scale manufacture of microstructured silicon substrates.

## 1. Introduction

Organic light-emitting diodes have numerous distinct advantages, such as rapid reaction time, great contrast, low power consumption, and so on [1,2,3,4]. Because they can be applied to TVs, mobile phones, and other full-color flat-panel displays and solid-state lighting sources, they have received a great deal of attention from academics and the business community. For a long time, research on improving the performance of OLED devices has mostly concentrated on the basic topic of how to effectively enhance device luminosity efficiency. Enhancing the light extraction efficiency of OLED devices, in addition to enhancing their internal quantum efficiency, is an efficient technique to enhance the luminosity efficiency of OLED devices [5,6,7,8,9,10]. Periodic micro-/nanostructure substrates may significantly increase the light removal efficiency of OLED devices [11,12]. However, many of the present technologies for creating micro-/nanostructure substrates are not suited for large-area preparation, which slows down the development of microstructure substrate OLEDs. Although electrochemical corrosion may be used to produce a vast range of microstructure patterns, it is often isotropic, making it difficult to regulate the direction of corrosion [13,14,15,16]. In this paper, we describe a method of electrochemically corroding silicon wafers that allows us to regulate the direction of corrosion to obtain the desired microstructure, which can be utilized to build a silicon-based microstructure substrate over a broad area. 

## 2. Experiment

**Figure 1:**
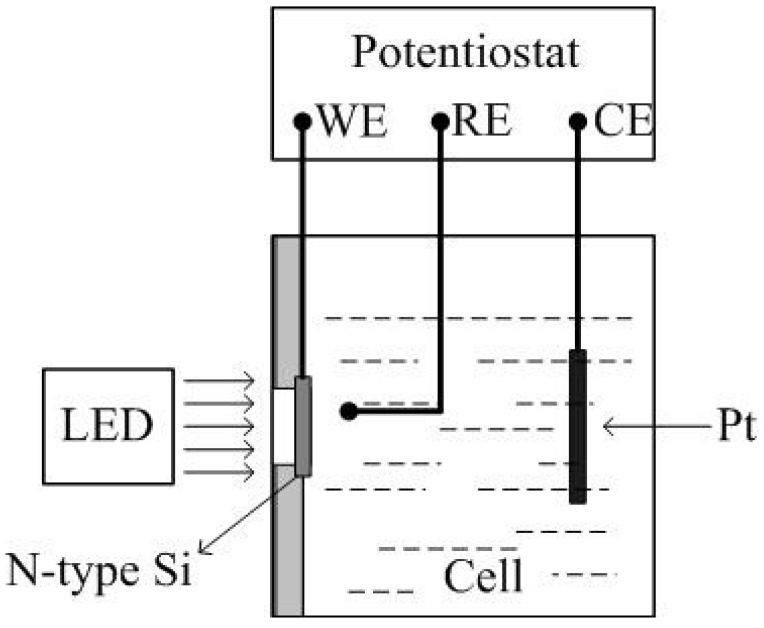
The microstructure substrate fabrication device.

Figure 1 depicts the microstructure substrate manufacturing device, an electrolytic cell with a side opening. The working electrode (WE) silicon was placed in the opening position by a sealing gasket, and was directly linked to the potentiostat via the ohm contact layer. The electrode (CE) is made of a 25 cm^2^ Pt wire, the reference electrode (RE) is made of a saturated calomel electrode, and the three-electrode system is controlled by a potentiostat (PARSTAT 2273 type). The etching solution is a HF acid solution that has been diluted with deionized water and has had a surfactant added. The thermostat bath keeps the temperature of the solution at room temperature (25 °C).

After receiving the patterned substrate, we cleaned the substrate and then utilized the thermal evaporation technique to grow 80 nm of Ag on the patterned substrate as the anode. Following that, a 60 nm N,N′-Bis(naphthalen-1-yl)-N,N′-bis(phenyl)benzidine (NPB) acting as the hole transport layer, a 70 nm Tris(8-hydroxyquinolinato)aluminum (Alq) acting as the emitting layer, and a 20 nm LiF and Ag layer acting as the composite cathode were evaporated [17,18,19,20,21,22]. All of the layers were prepared by thermal evaporation in a high-vacuum system (Shenyang Sida Vacuum Technology Research Institute, SD400B) with a pressure of less than 4 × 10^−4^ Pa. The deposition rate of the electrode was about 1 nm/s, and the deposition rate of the organic material was 1–2 Å/s. The deposition rate was monitoring by a film thickness monitor (SHANGHAI TAIYAO VACUUM TECHNOLOGY CO., LTD., FTM-V, Shanghai, China) The effective luminous area of the device was 1 mm^2^. After completing the OLED preparation of the patterned microstructure substrate, the current–voltage–brightness (J-V-L) test was completed using a computer-controlled Keithley2611 digital source table and a Konica Minolta CS-100A photometer. All of the tests were carried out at room temperature.

## 3. Results and Discussion

Because holes are minority carriers in n-type silicon, they must be generated by light or a stronger electric field during the electrochemical corrosion process. To produce the holes in the study, an LED was employed as the light source. Distinct wavelengths of light stimulate silicon to create distinct photocurrents under the same light power circumstances, indicating that the spectral response varies with wavelength.

**Figure 2:**
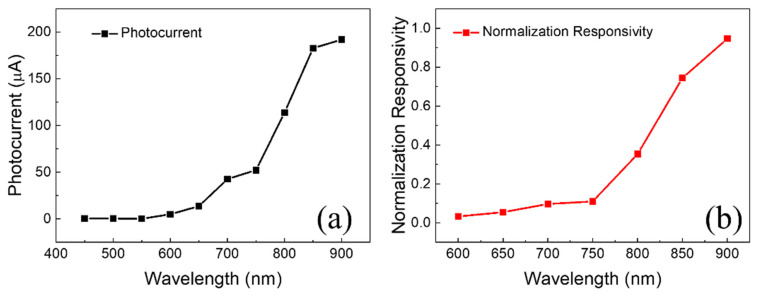
The wavelength responsivity curves of the electrochemical corrosion experiment. (**a**) Photocurrent wavelength characteristics, and (**b**) normalization responsivity wavelength characteristics.

The associated electrochemical corrosion photocurrent measured under different LED wavelengths is shown in Figure 2a. To decrease the dark current, the solution concentration was kept low (1 wt%) throughout the measurement, and the anionic surfactant was utilized. Figure 2b depicts the predicted relative spectral response curve of silicon photoelectrochemical corrosion. The spectral response rises dramatically with increasing wavelength, as illustrated in Figure 2b. For this experiment, the junction created between the silicon and the HF solution in photoelectrochemical corrosion of the n-type silicon was equal to a reverse-biased PN junction. When backlighting occurs, the created holes diffuse to the interface, and the silicon corrodes to make a photocurrent. In the experiment, the LED light source was placed on the back of the silicon, far away from the PN junction contact (the silicon wafer thickness was 400 m). Because the majority of the photogenerated holes are recombined and do not contribute to the current, the photocurrent value was minimal.

It is generally known that the absorption of silicon to light decreases as the wavelength of the light increases. As a result, the greater the wavelength of light, the deeper the depth of penetration into silicon. Because the LED is placed on the back of the silicon in this experiment, the longer wavelength light may penetrate deeper, bringing the holes created by excitation closer to the Si/HF contact and improving the spectrum response. We selected 850 nm as the LED emission wavelength after careful analysis.

**Figure 3:**
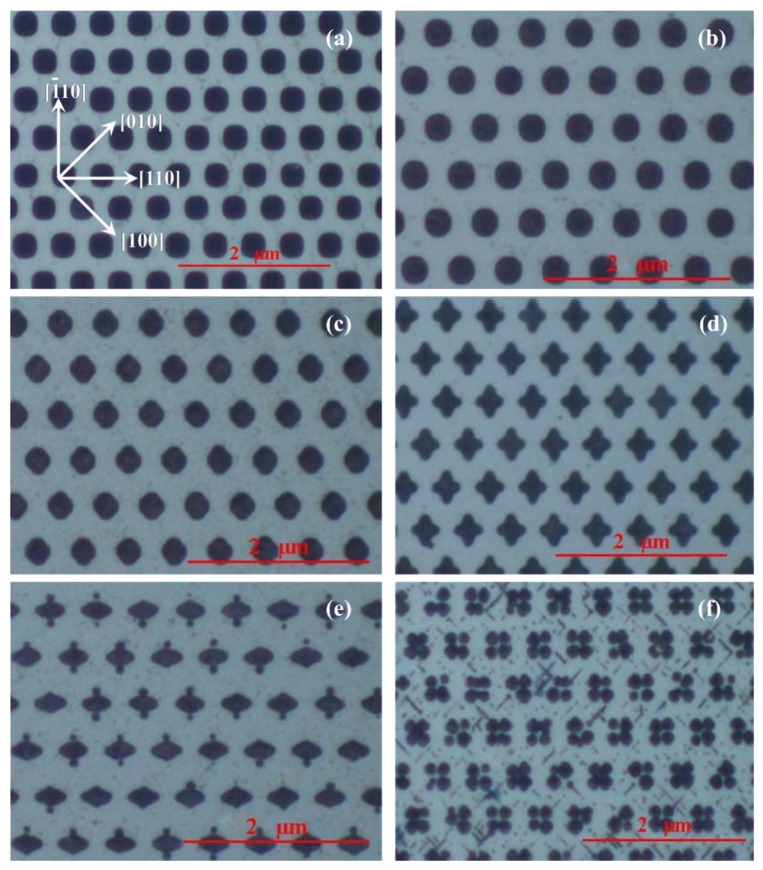
Microscopic photos of hole cross-sections under different voltages. (**a**) 0.6 V, (**b**) 1 V, (**c**) 1.5 V, (**d**) 3 V, (**e**) 5 V, and (**f**) 9 V.

Initially, we investigated the effect of the corrosion voltage on the patterning of the silicon substrate. To begin, the corrosion voltage was set to 0.6 V, 1 V, 1.5 V, 3 V, 5 V, and 9 V, with the corrosion current density set to 10 mA/cm^2^ and the corrosion time set to 1 h. The front of the sample was then ground and polished. As shown in Figure 3, the cross-sectional morphology of the hole was obtained using a metallographic microscope (JEOL, JSM-7500F, Beijing, China). As can be seen in Figure 3, when the voltage increases, the cross-sectional form of the hole progressively changes from square to round. When the voltage was increased to 3 V, the shape transformed into a four-pointed star. When the voltage was 5 V, the hole forks, and when the voltage was 9 V, it split into several smaller holes. The phenomenon is generated by the pores’ spatial layout and the unequal distribution of the electric field at the pores’ tips. The holes are first gathered near the tip in a process of macroporous silicon electrochemical corrosion, and the variation in the spacing around the tip leads to a difference in the supply of holes. The stronger the electric field, on the other hand, the more unequal the distribution of the holes at the tip. The combination of the two results in the experimental phenomena is depicted in Figure 3.

**Figure 4:**
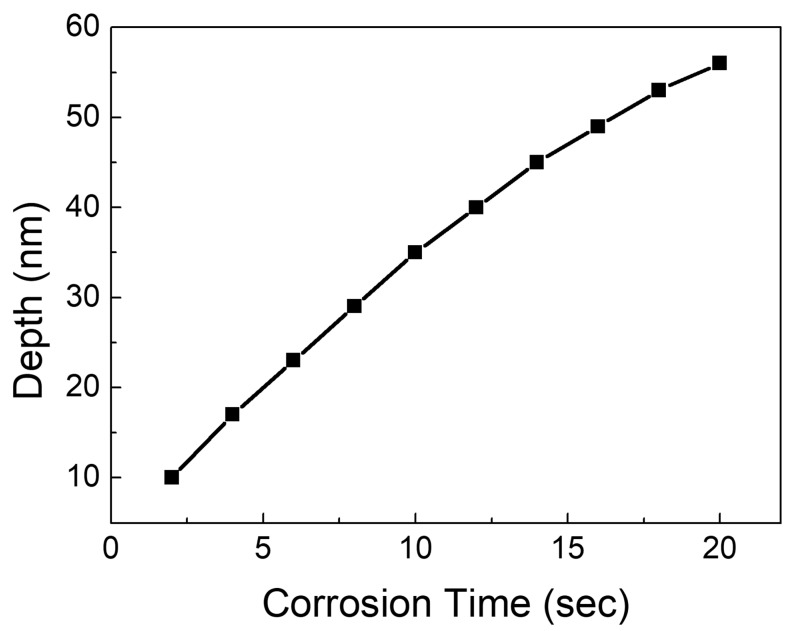
The relationship between the hole depth and the corrosion time.

The deeper the hole that produces the pattern on the silicon substrate, the longer the corrosion time. The link between hole depth and corrosion time is seen in Figure 4. When the corrosion voltage is fixed, the corrosion time mostly impacts the particle transport process in the solution. It had an effect on the transit of F-ions in this study. A sufficiently high corrosion voltage was utilized in this study to exclude the impact of F-ion concentration distribution on the experiment and meet the polarization requirement that the electrochemical reaction is exclusively affected by mass transfer. The corrosion depth of the hole was monitored every minute. As can be seen in Figure 4, the growth rate of the hole depth decreases as the corrosion duration rises. F-ions are primarily used in the electrochemical corrosion of silicon substrates to operate on the tip of the Si/HF interface, causing silicon atoms to mix with fluorine and form soluble substances, thereby enlarging the hole. The electrochemical reaction is mainly limited by mass transfer transport in the case of sufficient holes, and the F-ions in the solution are continuously transported to the tip of the hole under the action of the concentration gradient, but the reaction product is transported from the tip of the hole to the solution, which will hinder the transport of F-ions. As the hole deepens, more reaction products accumulate in the hole, and the transport of F-ions will produce greater obstacles. As a result, the corrosion process slows.

**Figure 5:**
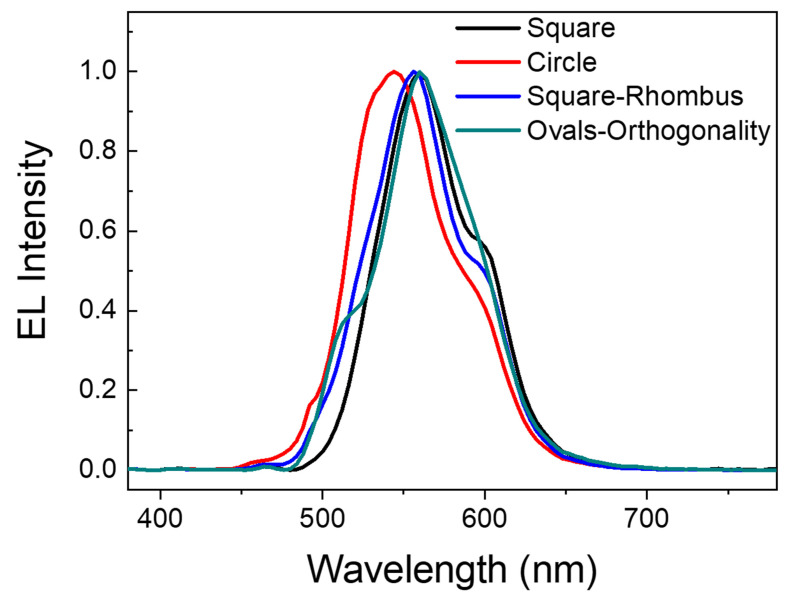
The simulation spectra of the OLEDs with a pattern of square, circle, square rhombus and oval orthogonality, respectively.

Patterns of varying shapes and depths may be produced on silicon substrates by manipulating the corrosion voltage and corrosion duration. The patterned silicon substrate may significantly increase OLED light extraction efficiency. We used the finite–difference time–domain approach to simulate light transmission in the patterned OLED to determine which design had the highest light extraction efficiency. We employed the Drude model to describe the dielectric coefficient of metals and materials, and the refractive index of the substance was fitted into the Drude model parameters using ellipsometer data (J. A. Woollam, M-2000UI, Shanghai, China). During the simulation, we used a periodic boundary condition for directions perpendicular to the pattern and a perfect matching layer (PML) to cut out other boundaries. We employed a modulated Gauss impulse with a center frequency inside the visible light spectrum of interest for the incident wave. At the completion of the computation, we isolated the transmission and reflection light components and utilized the Poynting vector ratio to represent transmission, reflection, and absorption. Figure 5 depicts the simulated spectra of the patterned OLED with the hole depth set at 20 nm. Figure 3 shows that as the voltage exceeds 5 V, the surface of the microstructure begins to reveal more flaws, making it unsuitable as an OLED substrate. As a result, we did not model these two categories. 

**Figure 6:**
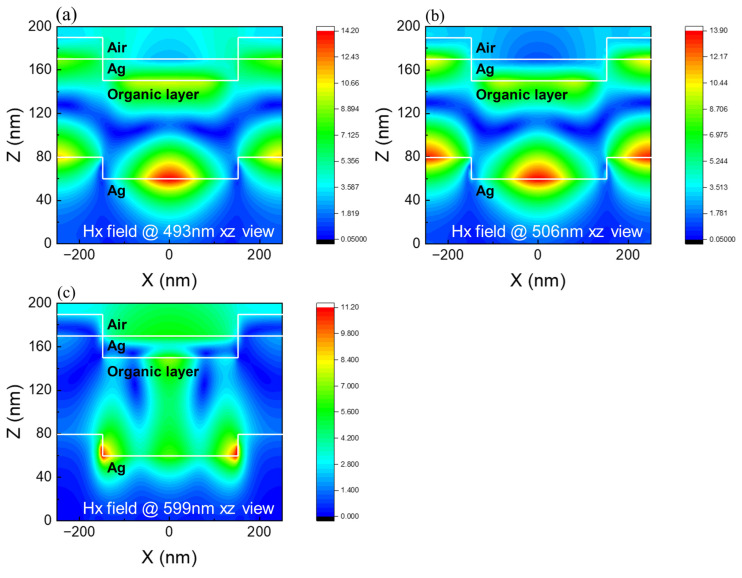
Distribution of magnetic field intensity in the corrugated OLEDs at wavelengths of incident polarized light of 493 nm (**a**), 506 nm (**b**), and 599 nm (**c**) in the XZ view, respectively.

Using the OLED simulation spectra with the circular pattern as an example, we can observe that there are three additional emission peaks at 493 nm, 506 nm, and 599 nm in addition to the Alq emission peak. To determine the cause of these extra peaks, we simulated and examined the field intensity in a circle-patterned OLED at an observation angle of 0°, as shown in Figure 6. Figure 6a,b show the 493 nm and 506 nm peak intensity distributions in TM mode. We can observe that the maximum field intensity occurs in the xz direction at the electrode/organic layer contact and in directions along the interface. This explains why the 493 nm and 506 nm peaks occur when the grating ignites surface plasma [12,20,21,22,23,24]. The waveguide mode, as shown in Figure 6c, has the maximum field strength in the xz directions, hence the 599 nm peak is created because the waveguide mode is stimulated. This suggests that the efficiency of light extraction from patterned OLEDs may be greater than that of planar OLEDs. Because the circumstances are the same for patterned OLEDs and planar OLEDs, except for the substrate pattern shape, we can anticipate which substrate pattern shape will provide superior performance based on the shape of the spectrum and the strength of the extra peaks. As a result, we can conclude that with the circular design, OLEDs will have a higher light extraction efficiency. 

**Figure 7:**
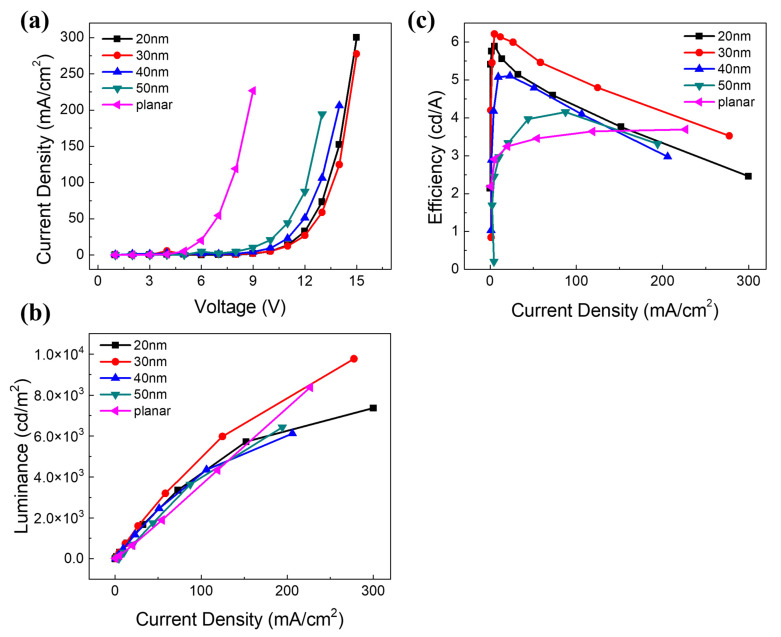
EL performance of the different corrugated-height and planar OLEDs. (**a**) Current density–voltage characteristics, (**b**) luminance–current density characteristics, and (**c**) efficiency–current density characteristics.

To put our theory to the test, we created planar and patterned OLEDs with varying hole depths. The substrate’s microstructures can be totally transmitted from the anode to the cathode. Despite the fact that the depths of the holes vary, and the area of the microstructures is considerably smaller than the luminous area of the OLED, the anode reflectivity of the patterned OLED is still extremely high overall and will not become diffuse owing to the existence of the microstructures. Figure 7 depicts the device performance curve. As seen in Figure 7, the current density of the patterned OLED rose greatly when compared to the control flat-panel device. This is because the pattern microstructure improved the device’s effective area. In addition, patterned OLEDs have superior brightness and efficiency when compared to flat-panel devices. The patterned OLED offers the best performance when the patterned hole depth is 30 nm, according to the performance comparison. At a current density of 100 mA/cm^2^, the patterned OLED has 1.67 times the brightness and 1.43 times the efficiency of a standard flat-panel device.

## 4. Conclusions

In this article, we apply the process of electrochemical corrosion to build a patterned silicon substrate. When the corrosion voltage intensities are varied, we can acquire six distinct patterned silicon substrates, and the power-on duration impacts the corrosion depth. We discovered that when the energized voltage is 1 volt and the energized time is 8 s, the resulting circle pattern silicon substrate OLED has the best performance: when the current density is 100 mA/cm^2^, its brightness is 1.67 times greater than that of the ordinary flat-structure OLED, and its efficiency is 1.43 times greater than the ordinary flat-plate-structure OLED. This electrochemical corrosion approach of producing patterned silicon substrates may provide a fresh direction for the future large-scale fabrication of microstructured silicon substrates.

## Data Availability

Data are contained within the article.
